# Predictive Value of Adiposity Level, Metabolic Syndrome, and Insulin Resistance for the Risk of Nonalcoholic Fatty Liver Disease Diagnosis in Obese Children

**DOI:** 10.1155/2018/9465784

**Published:** 2018-04-26

**Authors:** Zofia Prokopowicz, Ewa Malecka-Tendera, Pawel Matusik

**Affiliations:** School of Medicine in Katowice, Department of Pediatrics and Pediatric Endocrinology, Medical University of Silesia, Katowice, Poland

## Abstract

**Background:**

Nonalcoholic fatty liver disease (NAFLD) is the most common cause of chronic liver disease in obese children. Early diagnosis and treatment are essential for curing or slowing down the disease progression. The aim of the study was to assess the prevalence of NAFLD in this population and to identify anthropometrical and metabolic risk factors for NAFLD prediction and its development.

**Material and Methods:**

The study included 108 obese children. Anthropometric measurements, NAFLD diagnosis (based on ALT level and/or liver ultrasound), and metabolic syndrome (MS) components were assessed in all patients. Patients were divided into groups with and without NAFLD.

**Results:**

NAFLD was diagnosed in 49 (45%) patients with similar prevalence in boys (27; 55.10%) and girls [22 (44.9%), *p* = 0.089]. NAFLD patients had significantly greater waist circumference, WHR, and WHtR and significantly higher total cholesterol, triglyceride, and fasting insulin concentrations as well as higher glucose and insulin concentrations in 120 minutes of OGTT and higher HOMA-IR levels compared to group of patients without NAFLD. In NAFLD patients, MS was significantly more likely to be diagnosed than in group without NAFLD (40.82% versus 22.81%,  *p* = 0.04), but among the MS components only hypertriglyceridemia was significantly more frequently diagnosed in the group with NAFLD (*p* = 0.002). Among analysed parameters the best independent risk factor for NAFLD was fasting insulin concentration with the cut-off point = 18,9 uIU/ml (AUC = 0.829).

**Conclusions:**

NAFLD is a very common disease in obese children. NAFLD predictive risk factors include increased waist circumference, elevated WHR and WHtR, and elevated total cholesterol, triglycerides, and fasting insulin as well as elevated glucose and insulin concentration in the OGTT and HOMA-IR index. NAFLD increases the risk of potential cardiovascular complications expressed by diagnosis of metabolic syndrome. The best independent predictive risk factor for diagnosing NAFLD in obese children is fasting insulin > 18.9 uIU/ml.

## 1. Introduction

Nonalcoholic fatty liver disease (NAFLD) is the most common cause of chronic liver disease in children [1]. Due to the growing number of obese children the prevalence of fatty liver increases, and it was assessed in around one-third of clinical population [2]. NAFLD is defined as hepatic fat infiltration > 5% of hepatocytes, assessed by liver biopsy, after exclusion of excessive alcohol intake and other liver pathologies. In children NAFLD is, usually at the time of diagnosis, a simple steatosis, which is reversible, but also can initiate further stages of disease, such as nonalcoholic steatohepatitis (NASH), fibrosis, and cirrhosis. The pathogenesis of NAFLD and its progression are complex process, called multiple-hit theory combining environmental (dietary habits, physical activity [3, 4]), molecular (lipotoxicity [5]), endoplasmic reticulum stress [6], mitochondrial dysfunction [7], organokines effect [8, 9], genetic (polymorphism involved in the onset and progression of the disease), and other factors like dysbiosis [7, 10, 11].

NAFLD not only in adults but also in children is associated with severe metabolic disorders as hypertension, dyslipidaemia, increased risk of type 2 diabetes, metabolic syndrome, and cardiovascular diseases [12, 13]. For many years, NAFLD has been perceived as a hepatic consequence of insulin resistance, but recent studies have shown that fatty liver disease can precede with type 2 diabetes and metabolic syndrome and may even be a risk factor for their development [14, 15]. Due to biopsy limitations, hepatic steatosis is also diagnosed with biochemical tests like elevated alanine transaminase, or imaging methods, especially ultrasound. Despite the limitations, ultrasonography is characterized by high sensitivity (60–94%) and specificity (84–100%) for NAFLD detection [16]. Early diagnosis in the asymptomatic period and effective therapy implementation enable curing or, at least, slow down the progression of the disease.

The aim of the study was to assess the prevalence of NAFLD, as well as identifying additional predictive anthropometrical and metabolic risk factors for NAFLD development in the obese children.

## 2. Materials and Methods

### 2.1. Study Population

The study was prospective and included 108 children aged 6 to 18, hospitalized in our department between years 2012 and 2014, whose BMI exceeded 97 pc for sex and age, after informed consent. Children with acute infectious disease, chronic hepatitis of known etiology, using hepatotoxic drugs, or consuming alcohol were excluded from the study. A general medical examination, anthropometric measurements, laboratory tests, blood pressure measurements, and abdomen ultrasound imaging were performed in all subjects.

### 2.2. Anthropometric Evaluation and Body Composition

All anthropometric measurements were made in the morning, with fasting with empty bladder in the upright position. Subjects were barefoot and lightly dressed. Height and weight were measured to the nearest 0,1 cm and 0,1 kg, respectively, by using calibrated scale and Harpenden stadiometer. Waist and hip circumference were measured to the nearest 0,5 cm using standard technique with nonelastic tape, at the end of normal expiration. Waist circumference (WC) was measured at a point midway between the lower border of the ribs and the iliac crest, and hip circumference was measured at the widest part of the hip. BMI, waist-hip ratio (WHR), and waist-to-height ratio (WHtR) were calculated. Standard deviations scores for height and BMI were calculated using the LMS method based on Polish reference values [17].

Body composition was evaluated by bioelectrical impedance using the Tanita MC 980 MA device-multifrequency segmental analyser. Estimates of body composition were obtained from the equipped software. Patient stand barefoot on the marked electrodes and in straightened hands held the handles equipped with electrodes. The measurement of the tissue impedance through which the six-frequency (1 kHz, 5 kHz, 50 kHz, 250 kHz, 500 kHz, and 1000 kHz) and low intensity (<90 *μ*A) current was applied is painless and lasts for several seconds. Of the many variables, the following results were analysed: total body water in kg and % (TBW), fat mass in kg and fat%, fat-free mass in kg (FFM), muscle mass in kg, visceral fat in %, and basal metabolic rate in kJ (BMR). Moreover the following indicators were calculated: visceral fat to total body fat (visceral fat%/fat%), basal metabolic rate per kilogram body weight (BMR/kg), and standard deviations scores for fat percentage (fat% *Z*-score = 2  *∗* (fat%  − 50 pc fat%)/(98 pc fat%  − 50 pc fat%)) based on children reference values [18].

### 2.3. Liver Ultrasonography

Liver ultrasound examination was performed by two radiologists with Siemens Acuson Antares, convex transducer 5 MHz. Fatty liver was diagnosed on the basis of increased echogenicity of the liver parenchyma compared to the right kidney echogenicity.

### 2.4. Laboratory Assessment

Fasting venous blood sample was taken in the second day of hospitalization. Blood chemistry analyses, alanine transaminase (ALT), gamma glutamyltransferase (GGT), total cholesterol (Tch), high density lipoprotein (HDL), low density lipoprotein (LDL), triglycerides (TG), and bilirubin, were performed in hospital laboratory by using standard methods. Oral glucose tolerance test (OGTT; 1.75 g/kg body weight, up to 75 g) was performed in all patients. Glucose and insulin concentration were measured during fasting and in 120 minutes of the test. In this study, elevated ALT levels were identified for ALT > 35 U/l in children aged 3–11 years and ALT > 40 U/l in children aged ≥ 11 years. Hypertriglyceridemia was defined as fasting triglycerides ≥ 150 mg/dl, and low HDL cholesterol as fasting HDL < 40 mg/dl in children aged < 16 years and <40 mg/dl for boys or <50 mg/dl for girls aged > 16 years [19]. Impaired fasting glucose (IFG) was defined as fasting glucose ≥ 100 mg/dl.

### 2.5. Blood Pressure Measurements

Blood pressure (BP) was measured three times daily by the Korotkoff method using sphygmomanometer, on the right arm after 5–10 minutes of rest in the sitting position. The results were referred to Polish children reference values [20]. Patients with at least two measurements > 90 pc (high normal pressure) and patients with previously treated hypertension performed 24-hour ambulatory blood pressure monitoring using the Spacelabs ABP. Hypertension was diagnosed when the systolic blood pressure was above 95 pc for more than 25% of the measurements.

### 2.6. Insulin Resistance and Metabolic Syndrome (MS)

Insulin resistance was measured by homeostasis model assessment of insulin resistance (HOMA-IR) described by Matthews et al. [21], defined as follows: HOMA-IR = fasting glucose (mg/dl) × fasting insulin (uIU/ml)/405. The outcomes were referred to Caucasian obese children reference values [22].

We used the definition of the International Diabetes Federation (IDF) for the metabolic syndrome in children and adolescents [19]. The first criterion reached in all our patients was central obesity defined as WC > 90 pc for age and sex or above adult norms (>80 cm for women and >94 cm for men). If patients reached at least 2 of the other 4 criteria (TG concentration ≥ 150 mg/dl or hypolipemic treatment; HDL levels < 40 mg/dl (for girls > 16 years, HDL < 50 mg/dl); elevated blood pressure (systolic > 130 mmHg or diastolic > 85 mm Hg) or hypertension; fasting glucose ≥ 100 mg/dl; or type 2 diabetes) then the metabolic syndrome was diagnosed.

### 2.7. NAFLD Diagnosis

NAFLD was diagnosed based on ultrasound examination and/or elevated ALT.

### 2.8. Statistical Analysis

The statistical software (Statistica v. 12.5 and Microsoft Excel) was used in data analysis. Descriptive data were expressed as median, mean values, and standard deviations (SD) for continuous variables. Patients were divided into two groups: with and without NAFLD, which were then compared for anthropometric features, biochemical parameters, and metabolic syndrome components. Mann–Whitney* U*, ANOVA Kruskal-Wallis, Spearman rank correlation tests were used to compare continuous variables. Chi-square test was used for qualitative variables. The receiver operating characteristics (ROC) analysis was used to verify the characteristics of the independent variables and to assess an appropriate cut-off. Statistical significance was set at *p* value less than 0.05.

## 3. Results

**Figure 1:**
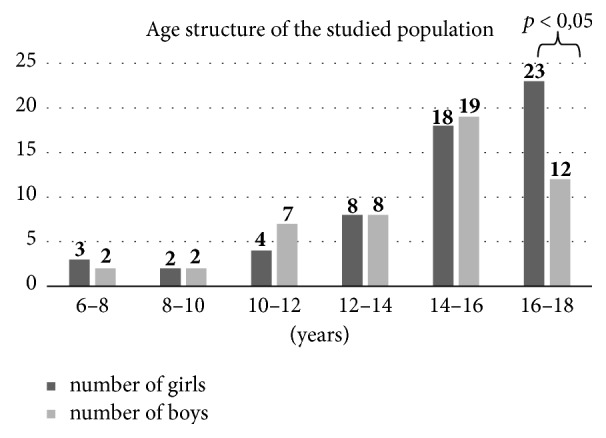
Sex distribution in particular age groups.

The study included 108 obese children (50 boys) aged 6 years and 2 months to 17 years and 10 months. Mean age was 14.24 ± 2.73 SD. Mean age of girls was 14.75 ± 2.79 SD and of boys 13.9 ± 2.64 SD. Girls were about one year older than boys, but the difference was not statistically significant (*p* = 0.074). The age distribution of the examined group is shown in Figure 1. The only statistically significant difference was observed in the 16–18-year age group, where girls predominated.  Table 1 gives the anthropometric and body composition characteristic of the study population stratified by gender. Males (*n* = 50) and females (*n* = 58) had similar BMI and height *Z*-scores while males had significantly higher mean WC and WHR. Among important anthropometrical data for obesity diagnosis, parameters of body composition males had significantly higher fat% and fat% *Z*-score; however no difference in visceral fat/fat% was observed.

### 3.1. NAFLD Prevalence

NAFLD was diagnosed in patients with hyperechogenic liver on ultrasound and/or elevated ALT concentration. Ultrasonographic features of fatty liver were diagnosed in 34 patients (16 girls and 18 boys) and elevated ALT in 30 patients (8 girls and 22 boys). In 15 patients (2 girls and 13 boys) both were reported. Based on proposed criteria, two groups were identified: (I) NAFLD patients (*N* = 49; 45.4%) and (II) non-NAFLD patients with normal liver and normal ALT concentration (*N* = 59; 54.6%).

NAFLD was diagnosed more often in boys than in girls; however the difference was insignificant (55.1% versus 44.9%; *p* = 0.089).

### 3.2. Anthropometry and Body Composition

NAFLD patients did not differ significantly from patients without NAFLD in age, height, body weight, BMI, or BMI *Z*-score, while NAFLD was associated with a significantly greater waist circumference, WHR, and WHtR. Body composition analysis did not show statistically significant differences between groups in key obesity parameters such as fat mass, fat%, fat%  *Z*-score, or visceral fat%. Groups differed significantly only in fat-free mass (FFM), total body water (TBW), and basal metabolic rate (BMR); however BMR per kilogram remained similar in both groups. The comparison of the two groups in terms of basic anthropometric and body composition parameters is presented in Table 2.

### 3.3. Biochemical Analysis

Of the biochemical parameters, the serum concentration of total cholesterol, triglycerides, fasting insulin, glucose, and insulin in 120 minutes of OGTT and HOMA-IR were significantly higher in patients with NAFLD.

NAFLD was significantly more often diagnosed in patients with HOMA-IR exceeding reference values [22] than in children with the normal range of HOMA-IR (79% versus 28%, *p* = 0.00 for HOMA-IR > 90 percentile; 85% versus 15%, *p* = 0.00 for HOMA-IR > 97 percentile). A detailed summary of the biochemical parameters is presented in Table 3.

**Figure 2:**
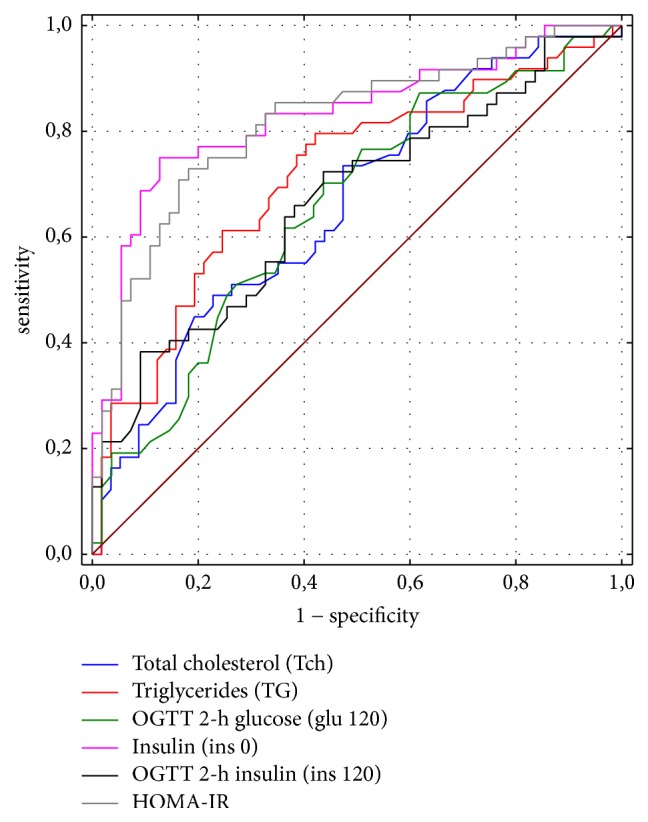
Comparison of ROC curves for parameters significantly differentiating patients with NAFLD from non-NAFLD patients.

The ROC analysis was performed on variables that differentiated NAFLD patients from non-NAFLD ones. Based on the area under receiver operating curves (AUC) we established a threshold of each variable that showed the highest accuracy for identifying children at high risk of NAFLD.  Figure 2 shows a comparison of the ROC curves for the evaluated parameters. The largest area under the ROC curve was obtained for fasting insulin (AUC = 0.829, 95% CI 0.746–0.911) and for HOMA-IR (AUC = 0.817; 95% CI 0,733–0,901). The optimal cut-off point of the insulin level for diagnosing NAFLD was 18,9 uIU/l with the sensitivity of 75% and specificity of 87,3%. The performance of these variables in NAFLD prediction is summarized in Table 4.

### 3.4. Metabolic Syndrome

In the study population 34 (31.48%) patients were diagnosed with metabolic syndrome (MS). Of the metabolic syndrome components abdominal obesity and hypertriglyceridemia were most often recognised. MS was diagnosed significantly more frequently in NAFLD group than in patients without fatty liver (40.82% versus 23.73%, *p* = 0.04), but of the MS components only hypertriglyceridemia was significantly more often diagnosed in NAFLD patients. The frequency of the MS and its components in the study population is presented in Table 5.

## 4. Discussion

We diagnosed NAFLD on the basis of elevated ALT and/or presence of steatosis on ultrasound. Both of these criteria are often used in noninvasive diagnosis of fatty liver disease. High sensitivity (60–94%) and specificity (84–100%) of ultrasonography make this examination suitable for screening [16]. Alanine aminotransferase (ALT) concentration due to wide availability and low cost is the basic marker used in liver diseases screening, including NAFLD. Interpretation difficulties are caused by different cut-off points proposed by each investigator like ALT > 52 U/L for girls and >72 U/L for boys in the Brazilian study [23], ALT > 50 U/L the in the Wiegand et al. study [24], or ALT > the laboratory standard in most authors' studies. On the other hand, we should be aware of low sensitivity of ALT at standard cut-off point (45 U/l); despite liver steatosis confirmed by imaging studies, in most pediatric patients, ALT levels remain normal. Therefore, a number of population studies suggest introducing new, sex-specific thresholds for ALT [25]: in children ALT < 25.8 IU/l for boys and ALT < 22.1 IU/l for girls in American study [26], ALT < 33 IU/l for boys and <25 IU/l for girls in Korean study [27], or ALT < 30 IU/l and <21 IU/l in Iranian study, respectively [28].

In this study we diagnosed NAFLD in 45% of patients. Such a high prevalence is caused by both group selection (obese children hospitalized for obesity) and diagnostic criteria. Fatty liver on ultrasound was reported in 34 (31.48%) patients, and elevated ALT levels were reported in 30 (27.78%) patients.

The authors of meta-analysis from 2015 [2], based on 56 clinical trials, assessed the prevalence of NAFLD in obese children on 34.2% with a prediction range from 5.2% to 83.1%. Researchers, however, emphasize significant differences in the methodology, diagnostic criteria, or accepted standards for the laboratory tests results. With the lowest frequency NAFLD was diagnosed in 2,3% patients by Brazilian researchers [23], which is caused by the inclusion criteria: abdominal obesity defined as WC > 75 pc and significantly higher ALT (52 U/L for girls and ALT > 72 U/L for boys) as well as ethnic differences (only 29% of the children were Caucasian). As much as 83.1% of patients were diagnosed with NAFLD in the American study [29]; however, the group consisted of children who were qualified to bariatric surgery. The mean prevalence of NAFLD diagnosed on ultrasound in the previously cited meta-analysis was 41%, while that diagnosed on the basis of elevated alanine aminotransferase was only 13.7% [2].

Males have higher prevalence of NAFLD than females, which is confirmed by most studies [2, 24, 30], but not in the Italian study [31] where NAFLD was similar in both sexes (58% of boys and 46% of girls, *p* = 0.31). However, in that study prepubertal population was analysed, which was significantly younger than in other studies. In our study prevalence of NAFLD was similar in males and females, which could have come from the sex distribution in age groups (there were significantly more girls in the 16–18 age group, *p* < 0.05).

In our study NAFLD patients were similar to non-NAFLD ones in age and basic anthropometric parameters such as height, weight and even BMI, and BMI *Z*-score. There was also no difference in the most important parameters of the body composition (fat mass, fat%, fat%  *Z*-score, or visceral fat%). Children with NAFLD had only significantly higher fat-free mass, total body water content, and BMR (but not BMR/kg); however, these parameters, due to their weight, age, or gender dependence, have little diagnostic value.

The only anthropometric parameters discriminating NAFLD patients from non-NAFLD, in our study, are waist circumference (WC) and the indicators associated with WC, WHR and WHtR. These are important, practical pieces of information indicating that BMI, even referred to reference values, is not sufficient for obese patients, while WC, which is still not a routine medical examination, may be an effective tool for detecting patients at risk of metabolic disorders, including NAFLD. Many studies have shown that waist circumference may be used in central obesity screening, cardiovascular risk assessment in children [32], and NAFLD diagnosis [33–35].

Similar results were obtained by Italian and Taiwanese researchers who did not observe differences in BMI and body composition between the groups with and without NAFLD, but waist circumference was significantly higher in NAFLD group [31, 34]. Denzer et al. also observed significantly greater waist circumference in patients with NAFLD (mean 111 cm versus 101 cm in patients without steatosis, *p* < 0.0001) but, in contrast to our results, NAFLD patients had significantly higher BMI-*Z* score (mean 2, 78 versus 2.56 in non-NAFLD, *p* < 0.0001) [30].

Considering the complexity of links between NAFLD, insulin resistance (IR), and type 2 diabetes, it is extremely difficult to find out whether NAFLD is the cause or the effect of insulin resistance. For many years, NAFLD has been treated as the hepatic consequence of IR, but recent studies suggest that hepatic steatosis may precede type 2 diabetes and metabolic syndrome and may even be a risk factor for their development [14, 15].

In our survey in diagnostics of carbohydrate metabolism fasting glucose remained useless, which was confirmed in other studies [31, 36]. Significant differences between groups were observed in fasting insulin concentration and glucose and insulin concentration in 120 minutes of OGTT. In our study, the highest diagnostic value for detecting NAFLD achieved fasting insulin with AUC = 0.829, and the cut-off point of 18.9 *μ*IU/ml with 75% sensitivity and 87.3% specificity diagnosed NAFLD. Studies in obese patients [31, 36, 37] confirm correlation of fasting insulin concentrations with NAFLD diagnosis; however there are no clear standards for this parameter, which makes it difficult to apply. In Shashaj et al. study [22] mean fasting insulin concentration in obese patients was 13.8 *μ*IU/ml ± 9.4, while in Pacifico et al. it was 10.6 *μ*IU/ml in obese children without steatosis and 15.6 *μ*IU/ml in obese children with NAFLD [36]. Much higher mean concentration of fasting insulin was observed by Korean researchers, 15.1 *μ*IU/ml ± 6.0 in obese children without NAFLD and 24.8 *μ*IU/ml + 14.6 in children with NAFLD [37]. These results are comparable to our study (12, 86 *μ*IU/ml ± 6.76 in children without steatosis versus 28.48 *μ*IU/ml ± 21.53 in children with NAFLD).

NAFLD patients had significantly higher HOMA-IR values compared to non-NAFLD ones. Similar observations have been made by Denzer et al. [30] and D'Adamo et al., who confirmed that insulin resistance indexes were significantly associated with NAFLD independently of BMI [31]. In contrast Gökçe et al. concluded that neither mean HOMA-IR values nor the prevalence of insulin resistance was higher in NAFLD group [12].

It is proven that insulin resistance increases in obese patients; therefore separate standards are developed for the population of obese children [22]. Authors of this study emphasize that in obese children HOMA-IR > 75 pc is associated with an increased cardiometabolic risk defined as at least one of the following: hypercholesterolemia, hypertriglyceridemia, reduced HDL levels, and ALT > 40 U/l. They have also observed that HOMA-IR > 3.42 (AUC = 0.71) with 48.8% sensitivity and 81.3% specificity identifies cardiovascular risk in obese patients. Referring our results to the proposed standards [22] we proved that NAFLD is significantly more common in patients with increased HOMA-IR compared to obese patients with normal HOMA-IR (79% versus 28%, *p* = 0.00 for HOMA-IR > 90 pc; 85% versus 15%, *p* = 0.00 for HOMA-IR > 97 pc), and HOMA-IR > 4.089 is a good indicator of NAFLD (AUROC = 0.817, sensitivity = 70.8%, specificity = 83.6%, and 95% CI = 0.733).

Metabolic syndrome (MS) in adult patients increases the risk of cardiovascular disease. In the pediatric population such conclusions are not clear. Due to both lack of long-term follow-up of pediatric patients and different criteria for MS, some authors suggest that the prevalence of individual components of metabolic syndrome in the pediatric population would be more relevant for assessment of the cardiovascular risk than the diagnosis of metabolic syndrome [38, 39]. In the studied population, based on IDF criteria [19], 34 (31.48%) patients were diagnosed with MS, which is comparable to other studies using the same criteria (Strojny et al., 29% [40]). In contrast, Manco et al. diagnosed MS only in 10% of children with NAFLD, which may be because only 65% of the children in the study group were obese [35]. In our study, patients with MS were more likely to have NAFLD than patients without MS (60.6% versus 39.19%, *p* < 0.05), but no correlation was found between the number of metabolic syndrome criteria and the prevalence NAFLD (*p* = 0.052), which may be caused by the fact that only fasting glucose but not the insulin resistance is considered in the IDF criteria for MS.

## 5. Conclusions

NAFLD is a very common disease in obese children. NAFLD risk factors include increased waist circumference, elevated WHR and WHtR, and elevated total cholesterol, triglycerides, and fasting insulin as well as glucose and insulin concentration in 120 min of OGTT and HOMA-IR index. NAFLD increases the risk of potential cardiovascular complications expressed by diagnosis of metabolic syndrome. The best independent risk factor for diagnosing NAFLD in obese children is fasting insulin concentration > 18.9 uIU/ml.

In our study, we focused on the diagnosis of obese children in the context of metabolic disorders, especially related to obesity and fatty liver disease. The prevalence of NAFLD and other metabolic disorders in the study population indicates the need to improve diagnostics in obese children already at primary health care level. Simple diagnostic methods such as waist circumference measurement and fasting plasma insulin concentration may contribute to the early identification and prediction of patients at risk of NAFLD and other metabolic complications. Appropriate therapy and lifestyle change in these patients and their families will help to prevent the negative effects of obesity in the future.

## Figures and Tables

**Table 1:** Anthropometric characteristic of studied population.

*Variable*	*All (N* = 108)		*Males (N* = 50)		*Females (N* = 58)		*ttest*
	Mean	SD	Mean	SD	Mean	SD	*p value*
BMI *z*-score	2.40	0.48	2.36	0.47	2.43	0.50	0.463
height *z*-score	0.42	1.4	0.60	1.55	0.27	1.26	0.230
*WC (cm)*	*103.9*	*12.9*	*108.17 *	*12.06*	*100.16 *	*12.49*	*0.001*
Hip circumference (cm)	108	11.3	108.12	11.47	107.90	11.29	0.922
*WHR*	*0.96*	*0.07*	*1.0*	*0.06*	*0.93*	*0.07*	*<0.001*
WHtR	0.63	0.06	0.64	0.06	0.62	0.06	0.073
*Fat*%	*39.27*	*6.79*	*37.17*	*7.36*	*41.08*	*5.71*	*0.002*
*Fat*% *z-score*	*3.06*	*1.03*	*2.84*	*0.87*	*3.31*	*1.14*	*0.017*
Visceral fat/fat%	0.46	0.45	0.44	0.45	0.48	0.45	0.769
*BMR (kJ)*	*7851*	*1233*	*8681*	*1056*	*7135*	*878*	*<0.001*
*BMR/kg (kJ/kg)*	*92*	*13.94*	*96.4*	*13.7*	*88.5*	*13.17*	*0.003*

**Table 2:** Anthropometry and body composition. Comparison of patients with and without NAFLD.

*Variable*	*NAFLD (N* = 49)			*Patients without NAFLD (N* = 59)			*p* value (Mann–Whitney *U* test)
	Mean	SD	Median	Mean	SD	Median	
Age (years)	14.40	2.49	14.83	14.17	2.94	14.92	0.828
*Waist (cm)*	*107.70*	*12.44*	*107.00*	*100.95*	*12.60*	*100.50*	*0.017*
Hip (cm)	108.78	11.50	109	107.53	10.97	108	0.794
*WHR*	*0.99*	*0.06*	*0.98*	*0.94*	*0.08*	*0.94*	*<0.001*
*WHtR*	*0.65*	*0.06*	*0.65*	*0.62*	*0.06*	*0.62*	*0.041*
Height (cm)	166.10	11.95	166.40	162.70	12.41	161.50	0.127
Weight (kg)	92.22	22.78	89.00	84.53	19.74	84.80	0.126
BMI (kg/m2)	32.97	5.29	32.00	31.49	4.82	31.60	0.243
BMI *Z*-score	2.45	0.48	2.38	2.37	0.48	2.36	0.479
Height *Z*-score	0.53	1.44	0.53	−0.573	0.566	−0.573	0.566
Fat%	39.40	6.34	38.90	39.15	7.23	39.60	0.897

Fat mass (kg)	36.61	11.58	36.50	33.44	10.91	34.60	0.214
*FFM (kg)*	*55.62*	*14.01*	*54.60*	*51.08*	*12.68*	*48.90*	*0.047*
FFM%	60.61	6.35	61.07	60.83	7.23	60.35	0.879
Muscle mass (kg)	52.40	13.72	51.80	48.57	12.13	46.60	0.094
*TBW*	*40.72*	*10.26*	*40.00*	*37.38*	*9.28*	*35.80*	*0.047*
TBW%	44.33	4.67	44.70	44.53	5.28	44.20	0.919
Trunk fat%	35.39	7.08	35.00	34.84	8.07	35.80	0.886
Fat% *Z*-score	3.15	0.99	3.04	2.98	1.07	2.92	0.577
Trunk/total	0.89	0.06	0.88	0.88	0.07	0.89	0.986
*BMR*	*8158*	*1293*	*7880*	*7617*	*1144*	*7512*	*0.028*
BMR/kg	90.89	12.33	86.17	92.80	14.85	88.57	0.597

**Table 3:** Biochemical analysis. Comparison of patients with and without NAFLD.

*Variable*	*NAFLD patients (N* = 49)			*Patients without NAFLD (N* = 59)			*p* value
	Mean	SD	Median	Mean	SD	Median	(Mann–Whitney *U* test)
*Tch (mg/dl)*	*185.12*	*28.64*	*185.00*	*169.02*	*30.01*	*167*	*0.004*
HDL (mg/dl)	45.34	10.26	43.90	47.77	11.38	45.85	0.298
LDL (mg/dl)	103.16	25.69	99.90	96.26	28.34	62.03	0.153
*TG (mg/dl)*	*183.08*	*79.69*	*173.00*	*129.15*	*62.03*	*114.00*	*<0.001*

glu 0 (mg/dl)	90.88	10.00	90.00	88.95	7.74	88.50	0.403
*glu 120 (mg/dl)*	*122.04*	*22.68*	*121.00*	*109.91*	*19.84*	*109.00*	*0.007*
*ins 0 (μIU/ml)*	*28.48*	*21.54*	*23.15*	*12.86*	*6.76*	*12.00*	*<0.001*
*ins 120 (μIU/ml)*	*130.38*	*98.18*	*93.80*	*81.36*	*44.03*	*70.70*	*0.004*
*HOMA-IR*	*6.44*	*4.83*	*4.93*	*2.86*	*1.67*	*2.58*	*<0.001*

**Table 4:** Diagnostic value of analysed variables in NAFLD prediction.

Variable	Symbol	AUC (95% CI)	Cut point	Sensitivity	Specificity	SE
Total cholesterol	Tch	0.660 (0.557–0.764)	197 mg/dl	44.9%	80.7%	0.053
Triglycerides	TG	0.713 (0.614–0.813)	161 mg/dl	61.2%	75.4%	0.051
Glucose in 120 min of OGTT	glu 120	0.654 (0.548–0.759)	112 mg/dl	70.2%	56.4%	0.054
Fasting insulin	ins 0	0.829 (0.746–0.911)	18.9 *μ*IU/ml	75%	87.3 %	0.042

Insulin in 120 min of OGTT	ins 120	0.666 (0.559–0.772)	129 *μ*IU/ml	38.3%	90.9%	0.054
Insulin resistance	HOMA-IR	0.817 (0.733–0.901)	4.089	70.8%	83.6%	0.043

**Table 5:** Prevalence of metabolic factors in NAFLD and non-NAFLD group.

MS components	Total (%)	NAFLD patients (*N* = 49)	Patients without NAFLD (*N* = 59)	*p*
WC > 90 pc	108 (100%)	49 (100%)	59 (100%)	NS
TG > 150 mg/dl or hypolipemic treatment	49 (45.37%)	30 (61.22%)	19 (32.20%)	*0.002*
HDL < 40 mg/dl (girls > 16 years; HDL < 50 mg/dl)	39 (36.11%)	20 (40.82%)	19 (32.20%)	NS
Hypertension	21 (19.44%)	11 (22.45%)	10 (16.95%)	NS
IFG or diabetes 2	13 (12.04%)	6 (12.24%)	7 (11.86%)	NS

*MS*	*34 (31.48*%)	*20 (40.82*%)	*14 (23.73*%)	*0.04*

## Data Availability

The data that support the findings of this study are available from the corresponding author [Pawel Matusik], upon reasonable request.
